# Supplementary material for: From biorepositories to data repositories: Open-access resources accelerate early R&D and validation of equitable diagnostic tools
Source: PLOS Glob Public Health. 2023 Aug 15;3(8):e0002044. doi: 10.1371/journal.pgph.0002044 (PMC10426984; doi:10.1371/journal.pgph.0002044)
Supplement: S1 Text — (PDF) [file pgph.0002044.s001.pdf]

DocuSign Envelope ID: EB0658F5-AEDF-442A-945B-230417366052

Biorepository Governance Plan for PATH'S Washington COVID-19 Biorepository

IRBNetID 1584172

Version 2.2

July 10, 2020

Confidential & Proprietary: For PATH Internal Use Only

PATH

Mailing Address
PO Box 900922
Seattle, WA 98109 USA

Street Address
2201 Westlake Avenue
Suite 200
Seattle, WA 98121 USA

www.path.org

© 2020 PATH. All rights reserved.

Contents

DOCUMENT APPROVAL 1

VERSION HISTORY 1

GOVERNANCE PLAN OVERVIEW 2

I. Statement of Principles 2

II. Description of the Biorepository 2

III. Communication with Participants Regarding Biorepository 3

IV. Biorepository Sample Identification 4

V. Biorepository Governance: Specimen Oversight and Access........ 4

VI. Returning Results to Participants and Research Communities 5

VII. Ongoing Communication about Biorepository Activities 5

Document Approval

Author and Public Health	Signature	Date
Helen Storey	DocuSigned by: Helen Storey 017C25A92B1B4F3...	July 29, 2020
Project leader (RPM)	Signature	Date
David Boyle	DocuSigned by: David Boyle D8A1120D128E4D4...	July 24, 2020
Commercialization	Signature	Date
Neha Agarwal	DocuSigned by: Neha Agarwal 1A872707684F42C...	July 27, 2020
Repository manager	Signature	Date
Roger Peck	DocuSigned by: Roger B Peck 502600609EE0493...	July 24, 2020

Version History

Version # (Date)	Key Changes from Previous Version
**1.0 (4/10/2020)**	Originally approved BGP
**2.0 (5/7/2020)**	Added additional partnerships to appendices; included general criteria for accepting new samples; updated formatting
**2.1 (5/12/2020)**	Addressing ORA comments for approval
**2.2 (7/10/2020)**	Added language to general criteria clarifying BSL 2 only samples, additional appendices of sample providers<br>Added language to note that some clinical panels may also include demographic and/or clinical data<br>Updated and added appendices

Governance Plan Overview

I. Statement of Principles

As a strategic partner to multiple private and public stakeholders, including funders, manufacturers, governments, researchers, and low- and middle-income countries (LMICs), PATH is well positioned to coordinate the operation of a biorepository related to the urgent coronavirus disease (COVID-19) response. In addition to a focus on scientific rigor, confidentiality, and ethical considerations, the repository owners, managers, and users are committed to the transparency and global access required to make this resource a broad platform for the global good.

II. Description of the Biorepository

The goal of the COVID-19 biorepository is to store and share samples in a ethical and globally accessible manner in order to advance development and commercial availability of COVID-19 diagnostic tests. With the current global COVID-19 pandemic, many diagnostic tests are being developed, however few are meeting the needs to support LMICs, where the implementation of the available tests is challenged by the reduced financial, infrastructural, and logistical inputs which are necessary to provide widespread and accurate diagnosis of those infected with the virus. The repository will acquire and qualify a variety of common specimen types associated with COVID-19 infection. These samples are intended for distribution with groups interested in developing such tests.

General criteria for accepting new samples are as follows:

1) Samples from *human subjects research studies* may be accepted into this repository if the studies include:
   - Broad consent language that allows for long term storage and future research on collected samples.
   - Participants have consented to long term storage and future research on their samples.
   - PATH will not be provided with identifiable biospecimens. Identifiable specimens are those for which the identity of the subject is or may readily be ascertained by the investigator or associated with the biospecimen. Biospecimens are considered identifiable if labeled with (a) a code that links to the identity of individuals and the investigator has access to the linking code, (b) direct identifiers, such as name or medical record number, or (c) a combination of indirect identifiers (e.g., date of birth, diagnosis, date of discharge) that could allow re-identification.
   - The material provided to PATH meets criteria for use in a BSL-2 laboratory.
2) Samples from laboratories conducting clinical testing may be accepted into this repository if:
   - The samples were collected for clinical care.
   - The samples are otherwise being discarded.
   - The samples provided to PATH will not be identifiable per same definition above.
   - The laboratory has the authority to share discarded samples for R&D purposes.
   - The material provided to PATH meets criteria for use in a BSL-2 laboratory.
3) Samples from research laboratories manipulating infectious material of COVID-19 may be accepted into this repository if:

- The cultured infectious material has been rendered non-infectious using documented procedures approved by PATH scientists and manipulation was conducted in a BSL-3 laboratory.
- The sample from which the infectious material was derived continues to be de-identified and no part of the material is linked to an individual.
- The material provided to PATH meets criteria for use in a BSL-2 laboratory.

The following partnerships have been established with the biorepository as of July 10, 2020. Details on partnerships are described in appendices.

1) The Everett Clinic in Washington State (appendix 1).
2) Washington State Department of Health (WA DoH) (appendix 2).
3) University of Washington (UW), Department of Global Health (appendix 3).
4) University of Washington, Department of Microbiology (appendix 4).
5) Fida Laboratories, Seattle (appendix 5).
6) Northwest Pathology Laboratory (appendix 6).
7) Bloodworks Northwest (appendix 7).
8) Stanford University School of Medicine (appendix 8).
9) UW, Department of Global Health, follow on to appendix 3 (appendix 9).

The Appendices will be reviewed on an annual basis and updated to reflect the partners providing samples to this biorepository.

The types of samples that will be collected, stored, and shared for future research.

The types of samples to be stored in the biorepository may include swabs from the nasal area, throat, and tongue, as well as saliva. The COVID-19 positive samples may contain high levels of the virus and will support the development of a method to directly detect the virus, which is the best way to indicate if someone is infected or not. Blood products, such as plasma and serum, will also be collected to enable the assessment of the immune response to infection over the course of the disease, a key factor for assessing the performance of serological tests. Derived, non-infectious material may also be added to the biorepository (ie. cultured and inactived virus, purified viral proteins, or RNA).

The type of research that the biorepository will support through future use.

The goal of the biorepository is to provide qualified clinical samples to various groups to support diagnostic technology development and to assess the performance of tests to diagnose COVID-19. The biorepository may also support basic research into the human immune response to the disease, as there is little understanding of the immunology and serology associated with COVID-19 as it is a novel pathogen. PATH may also procure deidentified biospecimens from valuable suppliers such as blood banks, if the unique sample type is critical for the work of developing and validating needed COVID19 diagnostics. Biospecimens from the repository will not be sold to any recipients.

III. Communication with Participants Regarding Biorepository

PATH will not receive identifiable specimens and PATH has no role communicating information or results to individual patients. The information generated from the future research will be used for the

development and validation of new diagnostics and therapeutics and will have no value to individual patients regarding clinical care and management.

IV. Biorepository Sample Identification

Who will have access to samples and data, and for which purposes?

PATH will store samples in a secure, card access only laboratory. To transfer samples from the partner to PATH, an material transfer agreement (MTA) will be developed and signed by PATH and the partner. When transferring clinical discard samples to PATH, the partner will provide written approval (email allowed) of authority to transfer samples and ownership to PATH in place of an MTA. All stored samples and data may be shared with other groups (recipients) upon the signing of a PATH/recipient mutually agreed MTA. Where required by the partner and per the terms of the PATH/partner MTA, written approval will be obtained from the partner and/or the foundation prior to accessing or sharing their specimens.

Which identifiers, if any, will be kept with the samples?

Specimens will be labeled with a unique identifier that is provided by the partner suppling the samples. For longitudinal samples, an identifier will be used to link samples from the same individual but distinguish sample collection dates.

The oversight of the linking code (links to identifiers/samples), if applicable.

All linking codes will be maintained by the partner providing the samples and will not be available to PATH.

Whether there are links to clinical data. The data associated with the samples will include the sample type, diagnostic test result and the date of sample collection. When samples are obtained from human subjects research studies, some or all data collected in the study, such as demographic and clinical presentation information, will accompany the samples. No identifiable patient data will accompany the samples.

Under what circumstances, if any, participants will be re-identified. None

What tracking systems are in place to document/track where samples are going and inventories of available samples.

Storage and shipping logs will be used to track inventory and the shipping of samples. All samples that enter the PATH laboratory are logged in per our biorepository protocol (Document number P2117). Samples will be aliquoted and stored in labeled boxes in a dedicated -80°C freezer. Each aliquot will be stored in an individually labelled tube or receptacle and logged into an Excel spreadsheet that serves as the inventory. The tracking of materials will be logged via MTAs with recipient groups that receive materials. The amount of material requested will be logged into the signed MTA and the individual lots cleared from the inventory as samples are accessed.

V. Biorepository Governance: Specimen Oversight and Access

A. Ethical Oversight

All samples in the PATH biorepository will be deidentified. Future research using these specimens may occur by PATH researchers or by external recipients.

DocuSign Envelope ID: EB0658F5-AEDF-442A-945B-230417366052

For research by PATH researchers: PATH will provide ethical oversight, and future use of samples will first involve submission of an application to PATH's Research Determination Committee (for non-FDA regulated activities) or an application to PATH's Research and Ethics Committee (FDA regulated activities).

For research by external recipients: Ethical oversight will be led by the recipient institution, and the researchers will go through their institution's policies for use/receipt of stored biospecimens. If the recipient institution does not have such policies (smaller manufacturers or other industry partners), the process above for PATH researchers will be followed as applicable per REC policies.

B. Legal Oversight

All specimens will be licensed and legally transferred to PATH and legal oversight will be provided by PATH. Written approval from the Foundation and PATH will be obtained for accessing and sharing specimens. When required by the source provider of the biospecimens, and in accordance with the PATH/partner MTA, written approval from the provider will also be obtained. PATH will not send any samples or associated data related to the biorepository without first executing an MTA with the interested party (recipient), unless in the unique scenario in which the source provider is unable or unwilling to enter into an MTA for business reasons. In this scenario, PATH will address and agree to key provisions of the MTA via documented email with the source provider. PATH/Recipient MTAs for access and sharing of samples will address the following:

- The pertinent terms of the biorepository, including intended use of the materials.
- Access, and transfer adheres to applicable federal, state, and local laws and regulations.
- Reporting, publication, and acknowledgement requirements as dictated by the PATH-sourcing party MTAs

C. Scientific and Global Access Oversight

Scientific oversight will be provided by the PATH Principal Investigator (PI) as part of a committee of Scientific Officers comprising of two PATH scientific officers and one scientific officer from the Foundation. When required in the partner MTA, a scientific officer from the provider institution will also be included. Any future research with biospecimens will be done with majority agreement from and in collaboration with the scientific committee. The recipient's intended use of the materials, as well as technology readiness and quality practices, will be the focus of the review by the scientific committee. The aim of the scientific committee is to confirm researchers seeking access to the biorepository meet the guiding principles laid out in this document. Recipients who demonstrate capacity, capabilities, and a willingness to commercialize resulting products in LMICs will be prioritized.

VI. Returning Results to Participants and Research Communities

Will results be communicated to the participants that provided samples, in individual or aggregate form? If so, describe how/and under what conditions this will be done.

All specimens in the biorepository will be deidentified and no results will be shared back to individual participants in individual or aggregate form.

VII. Ongoing Communication about Biorepository Activities

The PATH biorepository team believes that it is critical to disseminate findings around COVID-19 given that there is little information on the pathobiology of COVID-19 and as a consequence

diagnostic tools are becoming available but with less significant scrutiny via United States Food and Drug Administration (FDA) Emergency Use Authorization (EUA) rather than more stringent authorization such as 510-k clearance. Therefore, it is key to disseminate any new scientific results to the public health and medical communities. Research results from use of the samples may be shared with PATH and the Foundation, and where appropriate made available through publications, presentations, conferences, and other resources for a variety of audiences at a national and international level.

Publications and presentations resulting from the research should carry an acknowledgement of "Washington COVID-19 biorepository, managed by PATH and contributed to by the following partners: [list of partners to be provided at time of publication]" as the source of samples. As a courtesy to contributors of biospecimens, results from future research may be shared with such collaborators prior to publication if allowed by terms agreed upon between the PI and other researchers using the specimens.
