# Supplementary material for: From biorepositories to data repositories: Open-access resources accelerate early R&D and validation of equitable diagnostic tools
Source: PLOS Glob Public Health. 2023 Aug 15;3(8):e0002044. doi: 10.1371/journal.pgph.0002044 (PMC10426984; doi:10.1371/journal.pgph.0002044)
Supplement: Attachment — Submitted filename: Response to reviewers May2023.docx [file pgph.0002044.s002.docx]

**RE: “From biorepositories to data repositories: open-access resources accelerate early R&D and validation of equitable diagnostic tools.”, manuscript ID PGPH-D-23-00417**

Dear Dr. Dan Kajungu, Academic Editor, PLOS Global Public Health

We appreciate your response and the time and attention given by the reviewers. The thoughtful critique and addressing the comments have improved the paper. Our responses are provided below in red.

**Reviewer(s)’ Comments to Author:**

Journal Requirements:

1. Please amend your detailed Financial Disclosure statement. This is published with the article. It must therefore be completed in full sentences and contain the exact wording you wish to be published.

a. State the initials, alongside each funding source, of each author to receive each grant.

We have added all authors’ initials to each of the two funding sources.

Please review your reference list to ensure that it is complete and correct. If you have cited papers that have been retracted, please include the rationale for doing so in the manuscript text, or remove these references and replace them with relevant current references. Any changes to the reference list should be mentioned in the rebuttal letter that accompanies your revised manuscript. If you need to cite a retracted article, indicate the article’s retracted status in the References list and also include a citation and full reference for the retraction notice.

To our knowledge, our reference list is complete and correct. No retracted papers have been cited. Please let us know if we are missing something.

Additional Editor Comments (if provided):

Authors can respond to the comment from reviewers.

**Reviewers' comments:**

Reviewer's Responses to Questions

1. Does this manuscript meet PLOS Global Public Health’s publication criteria? Is the manuscript technically sound, and do the data support the conclusions? The manuscript must describe methodologically and ethically rigorous research with conclusions that are appropriately drawn based on the data presented.

Reviewer #1: Partly

Reviewer #2: Yes

________________________________________

2. Has the statistical analysis been performed appropriately and rigorously?

Reviewer #1: N/A

Reviewer #2: N/A

________________________________________

3. Have the authors made all data underlying the findings in their manuscript fully available (please refer to the Data Availability Statement at the start of the manuscript PDF file)?

Reviewer #1: No

Reviewer #2: No

________________________________________

4. Is the manuscript presented in an intelligible fashion and written in standard English?

Reviewer #1: Yes

Reviewer #2: Yes

________________________________________

**5. Review Comments to the Author**

**Reviewer #1:** The authors of this manuscript (more a research methods paper, not a research paper per se) present a use case describing the considerations in developing a coordinated open-access specimen biorepository and data registry that can prioritize access and use for innovative research, development and validation of diagnostic tools. Distinguishing these efforts from other ‘academic biorepositories’ is, in part, the desire to support research applications amenable to translation to low and middle income countries, which in turn prompts special considerations in the implementation strategy. A critical point, made in several sections, is the importance of existing partnerships and thereby implicitly sustainability as a Gates Foundation-supported non-profit. More specific details on implementation and operations and on success in achieving its mission goals would be very helpful. The value of a capacity like PATH is an important message for the Global Health community,

STRENGTHS OF THE PAPER; Major Points for improvement

• The paper underscores the importance of pre-existing partners in addressing emergent public health emergencies. Through such relationships, organizations like PATH have developed the internal workflows and governance considerations that offer agility and speed in response.

o The partnerships established by PATH and reflected in this paper are quite diverse (academic health centers, non-profit labs, health departments, local clinics). What was the incentive to send their specimens to PATH? Articulating both the financial and non-financial incentives could help readers approach building similar relationships and infrastructure.

A large reason partners were willing and eager to participate was to contribute to the efforts to stop the Covid19 pandemic. Where samples would have been discarded anyway, donating to PATH was no financial loss to the partner. Where samples were prospectively collected by the partner, the reasons for that research were focused on understanding better the disease progression of Sars-Cov2 infection. Donating some of their samples to PATH was aligned with the purpose of the data collection activity. Additional text has now been added for clarity: “Samples were provided to PATH at no cost because partners were eager to contribute to stopping the Covid19 pandemic”.

In general, participants who donate data or biospecimens to science are making the choice from a place of societal benefit. Attribution is an important way of calling out their contribution, which is essential to most biomedical research.

• The authors emphasize the importance of diversity at every step, including specimens and data themselves as well as in the approaches to test and validate technology. Collaborations are key to accessing ‘hard to reach’ and vulnerable populations to make sure that research discoveries pertain to diverse populations. Were these relationships already in place prior to COVID-19?

The partnerships involved in the biorepository set up were preexisting through professional relationships and past work experience. PATH collaborates with a wide range of partners for ongoing projects and research. However, PATH does not commonly, if ever, collect samples from the USA for research to benefit other settings due to disease area focuses. More common for PATH is to collect samples from settings where donor priority diseases exist, to support development of new diagnostics for those settings. And this is accomplished through long standing and trusted partnerships across PATH’s 15 offices globally and the many other countries where PATH has ongoing projects. Additional text was added to give more detail on this: “To accomplish this goal, PATH maintains long-standing and trusted relationships with a wide range of partners including but not limited to entities in academia, national and subnational governments, nonprofit organizations, civil society organizations, global normative bodies, and industry.”.

• The authors identified key considerations in the establishment, implementation and sustainability of the resources (biorepository, data registry) – e.g., IRB, freezer space, MTAs, laboratory capacity (BSL2) –

o More specific insight into the branch point decisions that must be made in developing the structure if a group wished to implement at their site would be helpful. For example, are there different documentation requirements for accepting prospective research specimens vs remnant clinical specimens? is there a ‘go/no go’ determination of whether a new partner is onboarded? Are there ‘checks’ in assessing the rigor and consistency in collecting & handling specimens (the latter could be really important in downstream use and could represent variability in results)? Are there staffing considerations that stem from decisions about strategy and scale? Are there infrastructure considerations? More specificity on the “issues” which can be anticipated and the authors’ team approaches to safety, IRB, legal, MTA, etc. (lines 227-230) would be helpful..

This is a good question and likely to vary by context and function of the repository. For our work, ethical oversight was a key first consideration. There are differences in national and international standards for how clinical specimens can be used in research. For example, use of clinical discards may be handled differently in different countries. Working closely with experts on the topic is essential, and particularly in our case, ethical bodies who can guide responsible use of human specimens for commercial purposes. Beyond ethics, collaboration requirements and partnering was critical to ensure access to the samples was feasible, as all partners had legal and executive leadership that had to support the effort. Governance structures involved legal capability to transfer ownership of samples from provider to recipient, managing liability, and ensuring responsible use by recipients. Laboratory considerations included deciding how to store, process, ship, and assess the quality of samples (from reviewing collection methods, documentations, facility visits, etc.). And equally important was communications and dissemination, or no one would know it existed for use. A callout box has been added to the methods section to highlight some of these key domains necessary to launch the biorepository.

 To the latter point about “issues” – which of these were uniquely COVID-19 related (and may substantiate a “pandemic preparedness special consideration” subset) and which are generalizable to repositories/registries built outside of a public health emergency? How will these insights offer PATH and everyone that reads this paper greater agility to navigate the issues more effectively next time?

The main issue that was unique to Covid19 was the timeline we were working with, and trying to have the repository in place in weeks rather than months to start storing early samples. The checklist of key criteria are relevant to all repositories. The ethical oversight process may be similar across scenarios but each repository requires a new approval and governance plan for the specific context of the repository. Legal templates or communications processes could also be used repeatedly once set up, with some adjustments for each situation. However, different diseases may require different lab capabilities and BSL classification.

o The comment that “many partners did not have legal teams” (line 248) was especially meaningful and important as the U.S. research enterprise, broadly writ, is encouraged to expand the reach to underrepresented populations (e.g., via rural hospitals, federally qualified health centers, community locations).

We advocate for a right-sized approach to manage risk. High quality research and protection of human subjects is not negotiable but not all situations have the same potential for risk. Where risks are lower, we would like to err on the side of inclusiveness to make resources or collaborations accessible to more entities. Additional text was added for clarity: “This right-sizing approach also increases inclusiveness by supporting partners to fill gaps in capabilities, without sacrificing quality or safety.”

• The authors illustrate the flexible value proposition of their repository by pointing to end users that were able to “detect and resolve required modifications”. More detail would again be helpful as it would set up nicely the premise that “but for the use of PATH biospecimens, these technologic innovations would have been delayed, diminished or deterred”. Detail would elevate the claim from sweeping generalization to evidence-based insight.

One issue that manufacturers were able to “detect and resolve” using the samples from PATH was around ensuring tests could detect newer variants as the circulating variants changed in the population. The repository also consists of nasal swab eluant collected with a variety of types of transport media. The variety of repository specimens allowed manufacturers to assess the performance of their tests with an array of transport media. Additionally, many of the nasal eluate specimens in the repository were accompanied by data from standard clinical testing, including cycle threshold information on SARS-CoV-2 N, S, and ORF genes. These data allowed manufacturers to assess the performance of their tests with specimens containing lower concentrations of the target, helping them to identify if their tests had adequate performance for real-world specimens.

The text has been expanded to note these issues above and now reads: “Some partners who received specimens were able to detect and resolve required modifications for several of their diagnostic tests before deployment in a public health setting, for example ensuring detection of newer variants, and confirming performance of tests across transport media types and cycle thresholds.”

• PATH appears to prioritize research and innovation that will make diagnostic tools (and other clinical applications) more accessible to low and middle income countries. The core messages of this manuscript are just as relevant to rural settings in all countries and reinforce important considerations for the research infrastructure everywhere as our ability to conduct experiments at greater scale continues to grow.

This is a good point and we agree. Under resourced settings exist in every country. Additional words were added to the first sentence of the conclusion to highlight this. The sentence now reads: “The right diagnostic tools save lives if they are made widely accessible, especially in under-resourced settings across all countries.”

WEAKNESSES OF THE PAPER: Additional points for improvement

• Figure 1 provides little useful information and can be deleted.

The photos of the samples were included to help the reader visualize the work we are referring to. Sample collection and storage can look different by setting. We demonstrate what our setting looks like for readers to have a better context.

• The general framework of the first half of the paper is straightforward; however, the pivot (line 185) to applications (pulse oximeters) seems awkward. Can the authors make the transition from biospecimen to physiological measurement devices easier to grasp?

Thank you for noting the need for more clarity in this transition. The point we want to make is that clinical information can come in many forms. Traditionally, biospecimens were the only clinical sample repositories included. However, “clinical specimens” are rapidly evolving, and images, videos, and even audio files are being used in research to derive clinical information. Repositories will need to expand to continue to advance this research. The subsection headings have been revised to add clarity.

And with that, data repositories will bring new challenges like how to store and share these types of large data files most efficiently. From freezer storage to cloud storage, larger datasets are needed for machine learning advances, and those datasets will likely leverage multimodal data sources. A PPG waveform is one example of a data source that includes many data signals per second, and AI will be useful to find meaningful patterns in the signals. Similar to accelerating open access publications, there may be a role for donors to play in accelerating the access and benefits of open access repositories. For example, offsetting the costs of cloud storage or developing common platforms for researchers could make these global public goods more accessible. A new paragraph was added to the last subsection of the results to speak to this point.

It reads: “Another challenge that arises as we transition from freezer storage to cloud storage is how to store and share large types of data files most efficiently. Larger datasets are needed for machine learning advances, and those datasets will likely include multimodal data sources. A PPG waveform is one example of a data source that includes many data signals per second, and AI will be useful to find meaningful patterns in the signals. Similar to accelerating open access publications, there may be a role for donors to play in accelerating the access and benefits of open access data repositories. For example, offsetting the costs of cloud storage or developing common platforms for use by researchers, could make these global public goods more accessible and quickly.”

o COVID-19 is caused by the SARS-CoV-2 virus… COVID-19 can lead to hypoxemia. The latter is not the cause of the illness, but is symptomatic of it (line 186)

We have changed wording from cause to symptom. Thank you for the catch.

• Figure 2 needs a legend in order to be clear about the message intended.

The following figure legend has been added to improve clarity: “There are key considerations as clinical samples or data are transferred from collection to storage to users. This flow diagram maps details for a specific PATH-led study; however, the attributes are relevant to other repository efforts as well. Early planning for the sources, structure, and purpose of the repository help inform later decisions on execution of the work”.

o It is also not explained how the approach “ensures high-quality and responsibly collected data” – by what methods is this ensured? What metrics are used to determine quality? How are issues like missing data or interoperability addressed?

In this context, quality is defined as achieves its intended purpose, equivalent or superior to accepted standards, and meets expectations. By thinking through key issues in advance, a higher quality product is achieved. The figure title was reworded to note this is one example of a structure and now reads: “Example of an open-access data repository structure to support high-quality and responsibly collected data is available for future development of public health products.”

• In the context of the data registry description (and data repository of Figure 2), the absence of comment about data models (e.g., OMOP) and FAIR principles that ensure that data from multiple sources can be integrated meaningfully is surprising. This deserves comment.

Additional text was added noting that the data repository will follow FAIR principles, and why/how this will be done. It’s an important detail that we agree is helpful to call out to readers. The added text now reads: “Additionally, the data repository will follow FAIR Principles (Findability, Accessibility, Interoperability, and Reusability).(21) The structure will be developed to follow general data modeling rules that reflect best practices for creating effective and efficient data models. This will include considerations such as identifying the entities and relationships that need to be represented, choosing appropriate data types and constraints, and defining clear and consistent naming conventions so the dataset could be merged with a larger system if required.”

• Every biorepository of this nature has a limited number of samples. The authors mention a request process (63 asks, 45 approved), but there was little explanation of if/how the group prioritized requests to maximize/extend the lifetime of the repository. On what basis were ~1/3 of the requests rejected, if other than lack of mission alignment to address LMIC goals?

The reason was sometimes mission alignment but there were others that were more logistical in nature. A sentence was added to note some reasons for not approving access, and it reads as such: “Pre-approval, reasons for declining requests included a lack of inventory of the requested sample, misalignment between technology stage of development and value/scarcity of sample type, and the requesting organization not having a BSL-2 laboratory, which was a safety requirement. Post-approval, some requests were later “declined” due to an organization’s inability to meet legal terms around global access and international shipping restrictions, which in cases prevented import of samples.”

o Did mechanical issues like freeze/thaw cycles factor in the dissemination process?

Serum and plasma were processed, including preparation of aliquots, prior to freezing. In some cases, larger volume aliquots were prepared and processed to smaller aliquots, which were re-frozen. Also, nasal eluate specimens were received to PATH frozen, so these specimens were thawed and processed to aliquots before refreezing. Freeze/thaw cycles of all specimens are tracked through the specimen ID, and internal PATH testing indicated similar performance of nasal eluate specimens after the freeze/thaw cycles.

An additional sentence was added to clarify, which now reads: “Additionally, freeze/thaw cycles of any specimens was tracked through the specimen ID.”

• The specimens were required to be de-identified and remnant specimens (the largest fraction of the biorepository) would have very little accompanying metadata. So, the process for characterization of “qualified, pedigreed” samples is very unclear… did PATH collect information about familial health?

In this context, “pedigreed” refers to where they were obtained and how, in other words by highly experienced and reputable sources. No genetic testing was conducted. The word “pedigreed” is now removed to avoid confusion.

FORMATTING CONSIDERATIONS

• The authors use a lot of acronyms that are not defined (e.g., RDT, LMIC, ORA) which makes reading very difficult. The acronyms should be defined.

All the acronyms have been double checked to ensure they are essential and defined. Changes were made to remove or add detail where it was needed, hopefully improving the readability of the manuscript.

• For those unfamiliar with PATH, it reads like another undefined acronym at (its mission is not defined until line 70). The authors should acknowledge that PATH is a non-profit organization committed global health equity issues by fostering innovative and accessible solutions in line 23 and 70.

Thank you for this suggestion. Additional text was added to line 23 that reads: “To address these challenges and gaps, PATH, a global nonprofit, along with its partners…”. As well as at line 72 that reads: “PATH, a nonprofit organization committed to global health equity, enables product development for low resource settings by…”.

• The quote in the first sentence of the introduction does not have a ‘close quote’ demarcation, making it unclear how much of that sentence is attributed to the reference.

The close quote has been added. Thanks for the catch!

**Reviewer #2:** The work shares experiences and lessons learnt relevant for future development efforts of Biorepositories and some immediate outcomes. I commend the authors for the well written article. I have a few comments here below.

1. Ethics statement – Given that the ethical clearance was for the repository being developed, it would be more meaningful to present it as one of the key steps needed for the development of a Biorepository.

We revised this section to describe the early considerations around ethics oversight.

2. Lines 135-136- The authors state that “samples were packed, couriered directly to PATH and immediately cataloged and processed with universal precautions even during citywide lockdown”. They however do not state how they dealt with these restrictions to be able to go about their work. -these experiences are important to highlight to inform future work amidst similar pandemics.

We added more detail as to what some of the adjustments were due to the lockdown. The text now reads: “Some of the modifications to procedures that were implemented due to the lock-downs include alternative transportation logistics as city buses were disrupted, use of additional personal protective equipment, social distancing procedures to limit the number of people in the lab at one time as well as to space out individuals in the lab, and creating designated work groups to assist in contact tracing if an exposure occurred”.

2. Although the establishment leveraged on existing PATH resources, it would be important to indicate cost considerations and how these were managed. For example, how much of the total establishment costs, at least an estimate, were covered by existing infrastructure etc.

Existing PATH resources allowed access to the needed expertise in a timely manner, however it didn’t offset costs significantly. Some project costs included accessing the needed resources in terms of staff time (ethics, legal, laboratory, administration, finance), reagents and materials for sample processing, and shipping logistics. Some costs covered by existing infrastructure included laboratory work space and freezer space to start.

3. I find that the information on stakeholders and their roles have been presented in very broad terms. The governance plan provides some more overview but again this has its own appendices that are not accessible. I would have wanted to see more details on key stakeholders in the development process beyond collaborations, governance etc. for example who they were and what were their roles in the establishment of the repository.

We name the partners we worked with starting on line 110. The details of the partnership vary and are confidential, which is why the appendices are not shared. Additional text was added to highlight some of the differences. The additional text reads: “The details of the partnership also varied slightly across partners. Some differences include how the samples were obtained, data accompanying the samples, the population from where the samples came, and ongoing communication with the partner about biorepository activities”.

4. lines 185-208. There is a long narrative under the section “Advancing PPG-derived technology through COVID-19” -that describes this new technology and how relevant it is to COVID19 testing. I however, did not understand how it fits with the “experience for repository development” that can be learnt. I would think that it is diagnostic technology related work not repository development.

Thanks for noting the need for more clarity in this transition, similar to the first reviewer. Please see our response above. In summary, the point we want to make is that clinical information can come in many forms and “clinical specimens” are rapidly evolving, and images, videos, and even audio files are being used in research to derive clinical information. Revisions have been made to improve readability.

5. Finally, I also think that one of the early outcomes of the Biorepository development is the data and metadata being created that can be used by developers to train their machine learning algorithms. This perhaps should have been highlighted as one of the low hanging fruit from this effort.

We have not seen a role for the biospecimens in training machine learning algorithms for Covid19 yet. However, for the two other repositories we describe that are focused on images and videos, the aim is that developers will request access to the data to train or validate their algorithms. This work is early, and we do not have robust findings to report on yet, though we will in the future.

________________________________________

Thank you again for the opportunity to continue to improve our work through this revised submission. If there are any other questions or concerns, we are happy to address them. We hope to hear favorably regarding the publication of this manuscript soon.

Sincerely,

Helen Storey
